# A study of human Kallikrein-2 gene polymorphism with special reference to prostate cancer patients in India

**DOI:** 10.1186/1755-8166-7-S1-P4

**Published:** 2014-01-21

**Authors:** Rajesh Biswas, Shiv Deep Bansal, Kakoli Biswas, Shrawan K Singh

**Affiliations:** 1 Department of Zoology, Government Home Science College, Sector-10, Chandigarh, India; 2 National Centre for Human Genome Studies and Research, Panjab University, Chandigarh, India; 3 Department of Biotechnology, DAV College, Sector-10, Chandigarh, India; 4 Department of Urology, Postgraduate Institute of Medical Education and Research, Chandigarh, India

## Background

Prostate cancer is the third most common cancer among men in the world. The human kallikrein-2 gene (KLK2 gene) contains a mutant (T) for wild-type (C) allele substitution, resulting in a Trp to Arg change on the 250^th^ amino acid at the 784^th^ nucleotide of KLK2 cDNA. The reports on polymorphism in the KLK2 gene in relation to prostate cancer are contradictory among American and Korean population. Reports are not available regarding polymorphism in KLK2 gene in relation to prostate cancer in Indian population. The relationship between mutants (T) for wild-type (C) allele substitution of the KLK2, circulating human kallikrein-2 (hK2) levels and prostate cancer risk in Indian population has been examined.

## Materials and methods

The study was limited to 15 subjects that include seven patients with prostate cancer and six controls who were persons with no cancer. Peripheral blood samples were collected from subject and control. DNA was extracted from these samples using standard protocol. DNA was dissolved in 1X TE buffer and used for PCR reactions for the amplification of 322bp KLK2 gene. Polymerase chain reaction- Restriction fragment length polymorphism (PCR-RFLP) procedure was employed using MspI to detect this polymorphism. The enzyme recognizes the 4bp sequence 5ʹCCGG3ʹ at codon 226 of KLK2 gene. PCR-RFLP products ware separated in 2% agarose gel and viewed under Gel-doc system.

## Results

The median age of the eight subjects diagnosed for prostate cancer were 60, out of which 6 are found to be homozygous for CC allele of KLK2 gene, 1 was heterozygous having CT allele and 1 was homozygous for TT allele. From 7 subjects diagnosed negative for prostate cancer 2 were having the CT genotype and 5 were found to have the TT genotype.

## Conclusions

The study indicates a possible correlation among the C/T polymorphism of the KLK2 gene and circulating levels of hK2 and, in combination, may be predictive for prostate cancer. Though significant association has been found with small subjects, uses of allele marker for prostate cancer need further extensive study with large number of subject.

